# Bis(2,2′-bipyridine-κ^2^ *N*,*N*′)(3-methyl­benzoato-κ^2^ *O*,*O*′)zinc 3-methyl­benzo­ate–3-methyl­benzoic acid–water (1/1/2)

**DOI:** 10.1107/S1600536812034216

**Published:** 2012-08-08

**Authors:** Qiu-qi Ye, Jin-li Qi, Jian-li Lin

**Affiliations:** a Center of Applied Solid State Chemistry Research, Ningbo University, Ningbo, Zhejiang 315211, People’s Republic of China

## Abstract

The title compound, [Zn(C_8_H_7_O_2_)(C_10_H_8_N_2_)_2_](C_8_H_7_O_2_)·C_8_H_8_O_2_·2H_2_O, is comprised of a Zn^2+^ cation, two 2,2′-bipydine (bipy) ligands and one 3-methyl­benzoate anion (*L* ^−^) together with one uncoordinating *L* ^−^ anion, one uncoordinating H*L* mol­ecule and two lattice water mol­ecules. The Zn^II^ atom is coordinated by four N atoms of two bipy ligands and two O atoms from one *L* ^−^ ligand in a distorted octa­hedral geometry. Pairs of centrosymmetrically related complex mol­ecules form dimers *via* slipped π-stacking inter­actions between bipy ligands with an inter­planar distance of 3.470 (4) Å. The dimers are linked into supra­molecular chains along [111], *via* C—H⋯O hydrogen bonds. The uncoordinated *L* ^−^ anions, H*L* mol­ecules and water mol­ecules are connected with each other *via* O—H⋯O hydrogen bonds, forming chains between the metal complex chains and binding them together *via* C—H⋯O contacts. The resulting layers parallel to (010) are further assembled into a three-dimensional supra­molecular architecture through additional C—H⋯O inter­actions.

## Related literature

For general background to complexes with intriguing topological structures, see: Chen *et al.* (2010 ▶) and for complexes with potential applications in gas storage and separation, magnetism, luminescence and catalysis see: Bettencourt-Dias & Viswanathan (2006 ▶); Liu *et al.* (2006 ▶); Xu *et al.* (2010 ▶, 2011 ▶).
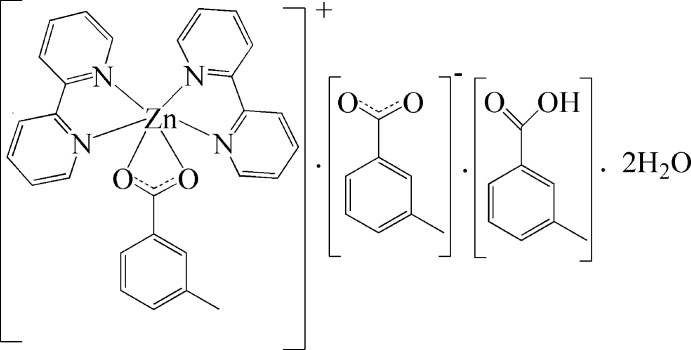


## Experimental

### 

#### Crystal data

[Zn(C_8_H_7_O_2_)(C_10_H_8_N_2_)_2_](C_8_H_7_O_2_)·C_8_H_8_O_2_·2H_2_O
*M* *_r_* = 820.21
Triclinic, 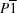

*a* = 12.690 (3) Å
*b* = 13.632 (3) Å
*c* = 14.493 (3) Å
α = 96.87 (3)°
β = 115.47 (3)°
γ = 110.96 (3)°
*V* = 1999.4 (13) Å^3^
*Z* = 2
Mo *K*α radiation
μ = 0.67 mm^−1^
*T* = 293 K
0.39 × 0.34 × 0.32 mm

#### Data collection

Rigaku R-AXIS RAPID diffractometer
Absorption correction: multi-scan (*ABSCOR*; Higashi, 1995 ▶) *T*_min_ = 0.769, *T*_max_ = 0.806
19803 measured reflections
9165 independent reflections
5744 reflections with *I* > 2σ(*I*)
*R*_int_ = 0.028

#### Refinement

*R*[*F* ^2^ > 2σ(*F* ^2^)] = 0.041
*wR*(*F* ^2^) = 0.147
*S* = 1.14
9077 reflections
514 parameters
H-atom parameters constrained
Δρ_max_ = 0.77 e Å^−3^
Δρ_min_ = −0.86 e Å^−3^

### 

Data collection: *RAPID-AUTO* (Rigaku, 1998 ▶); cell refinement: *RAPID-AUTO*; data reduction: *CrystalStructure* (Rigaku/MSC, 2004 ▶); program(s) used to solve structure: *SHELXS97* (Sheldrick, 2008 ▶); program(s) used to refine structure: *SHELXL97* (Sheldrick, 2008 ▶); molecular graphics: *ORTEPII* (Johnson, 1976) ▶; software used to prepare material for publication: *SHELXL97*.

## Supplementary Material

Crystal structure: contains datablock(s) global, I. DOI: 10.1107/S1600536812034216/mw2076sup1.cif

Structure factors: contains datablock(s) I. DOI: 10.1107/S1600536812034216/mw2076Isup2.hkl

Additional supplementary materials:  crystallographic information; 3D view; checkCIF report

## Figures and Tables

**Table 1:** Hydrogen-bond geometry (Å, °)

*D*—H⋯*A*	*D*—H	H⋯*A*	*D*⋯*A*	*D*—H⋯*A*
O4—H4*B*⋯O5^i^	0.86	1.63	2.492 (5)	175
O7—H7*B*⋯O5^ii^	0.86	2.45	3.028 (6)	125
O7—H7*C*⋯O8	0.88	2.14	2.938 (6)	151
O8—H8*B*⋯O6	0.88	2.10	2.973 (5)	178
O8—H8*C*⋯O6^ii^	0.85	2.05	2.871 (6)	163
C7—H7*A*⋯O2^iii^	0.93	2.45	3.234 (5)	142
C17—H17*A*⋯O1^iv^	0.93	2.44	3.297 (5)	152
C18—H18*A*⋯O8	0.93	2.47	3.280 (7)	146

Symmetry codes: (i) 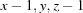; (ii) 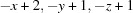; (iii) 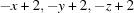; (iv) 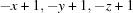.

**Fig. 1.**
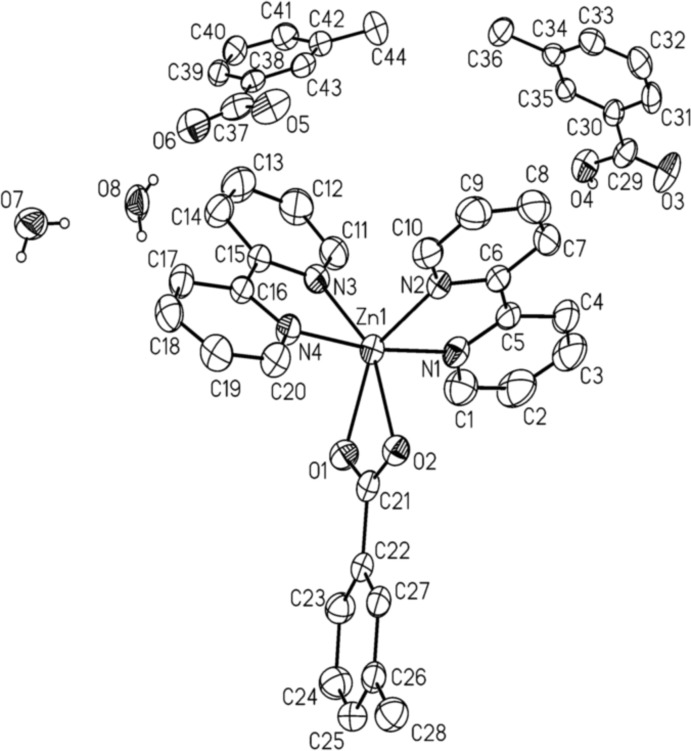
ORTEP view of the title compound. The displacement ellipsoids are drawn at the 30% probability level.

**Fig. 2.**
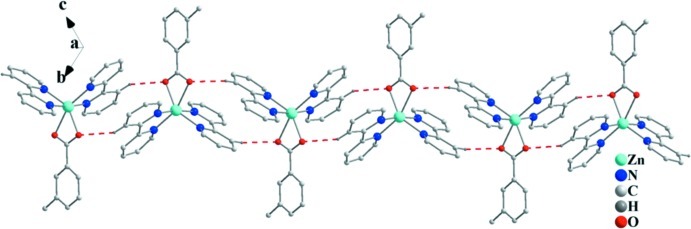
One-dimensional supramolecular chain along [111] formed by C7-H7A···O2 hydrogen bonds and π···π stacking interactions.

## supplementary crystallographic information

### Comment

In recent years, the design and synthesis of metal-organic frameworks (MOFs) have attracted considerable attention due to their intriguing topological structures (Chen *et al.*, 2010) and potential applications in gas storage and separation, magnetism, luminescence, and catalysis (Bettencourt-Dias *et al.*, 2006; Liu *et al.*, 2006; Xu *et al.*, 2010; Xu *et al.*, 2011). Our interest in self-assemblies of Zn^2+^ ions and 2,2'-bipyridine (bipy) with 3-methylbenzoic acid (HL = *m*-CH_3_-C_6_H_4_COOH), led to the preparation of [Zn(C_10_H_8_N_2_)_2_(C_8_H_7_O_2_)](C_8_H_7_O_2_).(C_8_H_8_O_2_).2H_2_O.

The asymmetric unit contains a Zn^2+^ ion complexed by two 2,2'-bipydine ligands and one 3-methylbenzoate anion (*L*^-^ = *m*-CH_3_-C_6_H_4_COO^-^) together with one uncoordinated *L*^-^ anion, one uncoordinated HL molecule and two lattice water molecules. The Zn ion is coordinated by four nitrogen atoms (N1, N2, N3, N4) of two bipy ligands and two oxygen atoms (O1, O2) from one *L*^-^ ligand in a tetragonally distorted octahedral geometry (Fig.1). The ligating atoms form a ZnN_4_O_2_ coordination environment with one carboxylate O2 atom, one pyridyl N3 atom in the axial positions and the other pyridyl N atoms and the other carboxylate O1 atom at the corners of the basal plane. The Cu-O and Cu-N bond lengths in the basal plane are in the range 2.114 (3) to 2.129 (3) Å and are slightly shorter than the axial Cu-O and Cu-N bond lengths (2.138 (3) and 2.308 (3) Å). Around the Zn^2+^ion the *cisoid* bond angles fall in the range 59.27 (9)-103.38 (10)°, and the *transoid* ones are 147.48 (10) and 171.36 (10)° thus exhibiting substantial deviations from 90° and 180° for an ideal octahedral geometry. The above observation indicates that the coordination geometry is a 4 + 1 + 1 type.

Two centrosymmetrically-related metal complexes molecules have bipy rings which are parallel with an interplanar distance of 3.470 (4) Å suggesting a slipped π···π stacking interaction. This together with weak, pairwise C17-H17A···O1 hydrogen bonds (Table 1) form dimeric units. Along the [111] direction the dimeric units are linked into one-dimensional supramolecular chains *via* pairwise C7-H7A···O2 hydrogen bonds (Table 1) and π···π stacking interactions with a distance of 3.455 (4) Å between the bipy rings which are not engaged in π···π stacking within the dimeric units (Fig.2). The uncoordinated *L*^-^ anions, HL molecules and water molecules connect with each other *via* O–H···O hydrogen bonds to form chains between the metal complex chains and connect with the latter *via* C8–H18A···O8 contacts (Table 1). The resulting layers are further assembled into a three-dimensional supramolecular architecture through additional C–H···O interactions.

### Experimental

1 mL of 1*M* aqueous K_2_CO_3_ solution was added to an aqueous solution of ZnSO_4_.7H_2_O (0.291 g, 1 mmol) to give a white precipitate from which SO_4_^2-^ anions were removed by centrifugation. The white precipitate was added to 20.0 mL of a H_2_O/CH_3_OH solution (1:1 *v*/*v*) of 3-methylbenzoic acid (0.271 g, 2.0 mmol). To the resulting solution was added 2,2'-bipydine (0.310 g, 2.0 mmol) whereupon the color of the solution became a light magenta and the pH was about 5. The solution was allowed to evaporate at room temperature for several days to give colourless block-shaped crystals.

### Refinement

H atoms bonded to C were placed in calculated positions and were refined using a riding model, with *U*_iso_(H) = 1.2 *U*_eq_(C). H atoms attached to O were placed in locations indicated by a difference Fourier synthesis and were refined using a riding model with *U*_iso_(H) values set at 1.2 *U*eq(O).

### Crystal data

**Table:**

[Zn(C_8_H_7_O_2_)(C_10_H_8_N_2_)_2_](C_8_H_7_O_2_)·C_8_H_8_O_2_·2H_2_O	*Z* = 2
*M**_r_* = 820.21	*F*(000) = 856
Triclinic, *P*1	*D*_x_ = 1.362 Mg m^−^^3^
Hall symbol: -P 1	Mo *K*α radiation, λ = 0.71073 Å
*a* = 12.690 (3) Å	Cell parameters from 19803 reflections
*b* = 13.632 (3) Å	θ = 3.0–27.5°
*c* = 14.493 (3) Å	µ = 0.67 mm^−^^1^
α = 96.87 (3)°	*T* = 293 K
β = 115.47 (3)°	Block, colorless
γ = 110.96 (3)°	0.39 × 0.34 × 0.32 mm
*V* = 1999.4 (13) Å^3^	

### Data collection

**Table:**

Rigaku R-AXIS RAPID diffractometer	9165 independent reflections
Radiation source: fine-focus sealed tube	5744 reflections with *I* > 2σ(*I*)
Graphite monochromator	*R*_int_ = 0.028
ω scans	θ_max_ = 27.5°, θ_min_ = 3.0°
Absorption correction: multi-scan (*ABSCOR*; Higashi, 1995)	*h* = −15→16
*T*_min_ = 0.769, *T*_max_ = 0.806	*k* = −17→17
19803 measured reflections	*l* = −18→18

### Refinement

**Table:**

Refinement on *F*^2^	Primary atom site location: structure-invariant direct methods
Least-squares matrix: full	Secondary atom site location: difference Fourier map
*R*[*F*^2^ > 2σ(*F*^2^)] = 0.041	Hydrogen site location: inferred from neighbouring sites
*wR*(*F*^2^) = 0.147	H-atom parameters constrained
*S* = 1.14	*w* = 1/[σ^2^(*F*_o_^2^) + (0.0452*P*)^2^ + 1.4221*P*] where *P* = (*F*_o_^2^ + 2*F*_c_^2^)/3
9077 reflections	(Δ/σ)_max_ = 0.001
514 parameters	Δρ_max_ = 0.77 e Å^−^^3^
0 restraints	Δρ_min_ = −0.86 e Å^−^^3^

### Special details

**Table:**

Geometry. All esds (except the esd in the dihedral angle between two l.s. planes) are estimated using the full covariance matrix. The cell esds are taken into account individually in the estimation of esds in distances, angles and torsion angles; correlations between esds in cell parameters are only used when they are defined by crystal symmetry. An approximate (isotropic) treatment of cell esds is used for estimating esds involving l.s. planes.
Refinement. Refinement of *F*^2^ against ALL reflections. The weighted *R*-factor *wR* and goodness of fit *S* are based on *F*^2^, conventional *R*-factors *R* are based on *F*, with *F* set to zero for negative *F*^2^. The threshold expression of *F*^2^ > σ(*F*^2^) is used only for calculating *R*-factors(gt) *etc*. and is not relevant to the choice of reflections for refinement. *R*-factors based on *F*^2^ are statistically about twice as large as those based on *F*, and *R*- factors based on ALL data will be even larger.

### Fractional atomic coordinates and isotropic or equivalent isotropic displacement parameters (Å^2^)

**Table:**

	*x*	*y*	*z*	*U*_iso_*/*U*_eq_	
Zn1	0.71782 (4)	0.74557 (3)	0.74582 (3)	0.05259 (13)	
O1	0.6240 (2)	0.79631 (18)	0.61420 (18)	0.0632 (6)	
O2	0.8392 (2)	0.88498 (18)	0.70427 (18)	0.0640 (6)	
N1	0.6910 (3)	0.8470 (2)	0.8486 (2)	0.0607 (7)	
N2	0.8825 (3)	0.7897 (2)	0.8995 (2)	0.0545 (6)	
N3	0.5646 (3)	0.5914 (2)	0.7187 (2)	0.0519 (6)	
N4	0.7404 (2)	0.6278 (2)	0.6566 (2)	0.0512 (6)	
C1	0.5911 (5)	0.8719 (3)	0.8168 (4)	0.0818 (12)	
H1A	0.5261	0.8421	0.7441	0.098*	
C2	0.5799 (6)	0.9392 (4)	0.8863 (5)	0.1013 (16)	
H2A	0.5085	0.9544	0.8617	0.122*	
C3	0.6764 (6)	0.9836 (4)	0.9928 (5)	0.1014 (17)	
H3A	0.6712	1.0295	1.0419	0.122*	
C4	0.7802 (5)	0.9601 (3)	1.0268 (3)	0.0820 (13)	
H4A	0.8472	0.9913	1.0988	0.098*	
C5	0.7856 (4)	0.8900 (2)	0.9540 (3)	0.0595 (9)	
C6	0.8922 (3)	0.8578 (2)	0.9814 (2)	0.0574 (8)	
C7	0.9989 (4)	0.8958 (3)	1.0859 (3)	0.0759 (11)	
H7A	1.0054	0.9431	1.1421	0.091*	
C8	1.0940 (5)	0.8625 (4)	1.1047 (4)	0.0896 (14)	
H8A	1.1652	0.8867	1.1743	0.108*	
C9	1.0846 (4)	0.7935 (4)	1.0215 (4)	0.0854 (12)	
H9A	1.1485	0.7703	1.0333	0.103*	
C10	0.9776 (4)	0.7598 (3)	0.9199 (3)	0.0688 (9)	
H10A	0.9712	0.7140	0.8628	0.083*	
C11	0.4801 (4)	0.5770 (3)	0.7542 (3)	0.0656 (9)	
H11A	0.4854	0.6396	0.7934	0.079*	
C12	0.3869 (4)	0.4753 (3)	0.7356 (3)	0.0797 (11)	
H12A	0.3295	0.4686	0.7609	0.096*	
C13	0.3801 (4)	0.3831 (3)	0.6788 (4)	0.0822 (12)	
H13A	0.3185	0.3127	0.6662	0.099*	
C14	0.4645 (4)	0.3951 (3)	0.6402 (3)	0.0653 (9)	
H14A	0.4602	0.3332	0.6010	0.078*	
C15	0.5560 (3)	0.5011 (2)	0.6610 (2)	0.0474 (7)	
C16	0.6491 (3)	0.5217 (2)	0.6214 (2)	0.0469 (7)	
C17	0.6421 (3)	0.4389 (3)	0.5502 (3)	0.0575 (8)	
H17A	0.5778	0.3660	0.5257	0.069*	
C18	0.7316 (4)	0.4660 (3)	0.5161 (3)	0.0669 (9)	
H18A	0.7284	0.4115	0.4683	0.080*	
C19	0.8248 (4)	0.5734 (3)	0.5529 (3)	0.0695 (10)	
H19A	0.8860	0.5930	0.5307	0.083*	
C20	0.8270 (3)	0.6521 (3)	0.6229 (3)	0.0621 (9)	
H20A	0.8910	0.7252	0.6480	0.074*	
C21	0.7311 (3)	0.8689 (2)	0.6291 (3)	0.0550 (8)	
C22	0.7267 (3)	0.9366 (2)	0.5545 (2)	0.0476 (7)	
C23	0.6095 (3)	0.9316 (3)	0.4820 (3)	0.0607 (8)	
H23A	0.5325	0.8853	0.4785	0.073*	
C24	0.6056 (4)	0.9951 (3)	0.4143 (3)	0.0705 (10)	
H24A	0.5264	0.9925	0.3661	0.085*	
C25	0.7197 (4)	1.0622 (3)	0.4187 (3)	0.0637 (9)	
H25A	0.7162	1.1048	0.3729	0.076*	
C26	0.8386 (3)	1.0681 (2)	0.4890 (3)	0.0527 (7)	
C27	0.8408 (3)	1.0045 (2)	0.5577 (2)	0.0511 (7)	
H27A	0.9201	1.0077	0.6066	0.061*	
C28	0.9624 (4)	1.1415 (3)	0.4927 (3)	0.0728 (10)	
H28A	0.9423	1.1788	0.4402	0.109*	
H28B	1.0003	1.0970	0.4767	0.109*	
H28C	1.0237	1.1954	0.5634	0.109*	
O3	0.3649 (3)	0.7905 (3)	0.0113 (3)	0.1100 (11)	
O4	0.3535 (2)	0.6528 (2)	−0.0970 (2)	0.0831 (8)	
H4B	0.2712	0.6332	−0.1361	0.125*	
C29	0.4130 (4)	0.7343 (3)	−0.0073 (4)	0.0706 (10)	
C30	0.5475 (3)	0.7524 (3)	0.0702 (3)	0.0576 (8)	
C31	0.6275 (4)	0.8414 (3)	0.1670 (3)	0.0736 (11)	
H31A	0.5955	0.8875	0.1853	0.088*	
C32	0.7526 (4)	0.8601 (3)	0.2344 (3)	0.0815 (12)	
H32A	0.8061	0.9199	0.2982	0.098*	
C33	0.8006 (4)	0.7927 (3)	0.2098 (3)	0.0713 (10)	
H33A	0.8862	0.8071	0.2572	0.086*	
C34	0.7241 (3)	0.7031 (3)	0.1155 (3)	0.0550 (8)	
C35	0.5977 (3)	0.6843 (2)	0.0478 (2)	0.0514 (7)	
H35A	0.5443	0.6235	−0.0152	0.062*	
C36	0.7767 (4)	0.6290 (3)	0.0881 (4)	0.0773 (11)	
H36A	0.7102	0.5718	0.0201	0.116*	
H36B	0.8021	0.5958	0.1435	0.116*	
H36C	0.8516	0.6719	0.0832	0.116*	
O5	1.1125 (3)	0.5888 (3)	0.7941 (3)	0.1124 (12)	
O6	1.0556 (3)	0.4608 (3)	0.6469 (3)	0.1140 (12)	
C37	1.0410 (4)	0.4929 (4)	0.7220 (5)	0.0855 (13)	
C38	0.9301 (3)	0.4148 (3)	0.7315 (3)	0.0568 (8)	
C39	0.8539 (3)	0.3053 (3)	0.6656 (3)	0.0617 (8)	
H39A	0.8716	0.2778	0.6150	0.074*	
C40	0.7520 (4)	0.2372 (3)	0.6752 (3)	0.0690 (10)	
H40A	0.7012	0.1632	0.6316	0.083*	
C41	0.7246 (4)	0.2779 (3)	0.7488 (3)	0.0718 (10)	
H41A	0.6541	0.2309	0.7533	0.086*	
C42	0.7983 (4)	0.3861 (3)	0.8160 (3)	0.0667 (9)	
C43	0.9022 (3)	0.4527 (3)	0.8068 (3)	0.0635 (9)	
H43A	0.9554	0.5257	0.8529	0.076*	
C44	0.7685 (6)	0.4310 (5)	0.8969 (4)	0.1070 (16)	
H44A	0.8303	0.5079	0.9357	0.160*	
H44B	0.7749	0.3899	0.9467	0.160*	
H44C	0.6813	0.4239	0.8598	0.160*	
O7	0.8097 (4)	0.1827 (3)	0.2299 (3)	0.1370 (15)	
H7B	0.8163	0.2131	0.1827	0.206*	
H7C	0.7934	0.2216	0.2699	0.206*	
O8	0.8568 (4)	0.3458 (3)	0.4133 (3)	0.1142 (12)	
H8B	0.9155	0.3783	0.4825	0.171*	
H8C	0.8661	0.3990	0.3874	0.171*	

### Atomic displacement parameters (Å^2^)

**Table:**

	*U*^11^	*U*^22^	*U*^33^	*U*^12^	*U*^13^	*U*^23^
Zn1	0.0613 (3)	0.0425 (2)	0.0479 (2)	0.02161 (17)	0.02557 (18)	0.01111 (15)
O1	0.0661 (15)	0.0479 (12)	0.0643 (14)	0.0136 (12)	0.0342 (12)	0.0186 (11)
O2	0.0690 (16)	0.0485 (12)	0.0554 (13)	0.0205 (11)	0.0217 (12)	0.0158 (10)
N1	0.078 (2)	0.0464 (14)	0.0648 (17)	0.0330 (14)	0.0386 (16)	0.0174 (13)
N2	0.0645 (17)	0.0384 (13)	0.0479 (14)	0.0186 (12)	0.0229 (13)	0.0122 (11)
N3	0.0627 (16)	0.0497 (14)	0.0472 (13)	0.0248 (13)	0.0316 (13)	0.0166 (11)
N4	0.0533 (15)	0.0439 (13)	0.0524 (14)	0.0198 (12)	0.0269 (13)	0.0107 (11)
C1	0.106 (3)	0.081 (3)	0.089 (3)	0.060 (3)	0.060 (3)	0.034 (2)
C2	0.153 (5)	0.092 (3)	0.133 (4)	0.086 (4)	0.103 (4)	0.056 (3)
C3	0.178 (5)	0.074 (3)	0.118 (4)	0.074 (3)	0.113 (4)	0.041 (3)
C4	0.137 (4)	0.051 (2)	0.072 (2)	0.037 (2)	0.069 (3)	0.0191 (18)
C5	0.088 (3)	0.0359 (15)	0.0550 (18)	0.0198 (16)	0.0435 (19)	0.0173 (14)
C6	0.071 (2)	0.0349 (14)	0.0487 (17)	0.0105 (15)	0.0272 (16)	0.0142 (13)
C7	0.086 (3)	0.0500 (19)	0.052 (2)	0.005 (2)	0.025 (2)	0.0125 (16)
C8	0.078 (3)	0.072 (3)	0.066 (2)	0.011 (2)	0.012 (2)	0.026 (2)
C9	0.072 (3)	0.076 (3)	0.084 (3)	0.028 (2)	0.022 (2)	0.036 (2)
C10	0.075 (2)	0.058 (2)	0.067 (2)	0.0316 (19)	0.029 (2)	0.0239 (17)
C11	0.083 (3)	0.066 (2)	0.062 (2)	0.032 (2)	0.050 (2)	0.0216 (17)
C12	0.088 (3)	0.080 (3)	0.083 (3)	0.027 (2)	0.061 (2)	0.031 (2)
C13	0.082 (3)	0.063 (2)	0.097 (3)	0.015 (2)	0.054 (3)	0.030 (2)
C14	0.067 (2)	0.0495 (18)	0.073 (2)	0.0202 (17)	0.0352 (19)	0.0175 (16)
C15	0.0498 (17)	0.0398 (14)	0.0431 (15)	0.0169 (13)	0.0195 (13)	0.0116 (12)
C16	0.0504 (17)	0.0414 (15)	0.0420 (15)	0.0216 (13)	0.0179 (13)	0.0119 (12)
C17	0.057 (2)	0.0505 (17)	0.0542 (18)	0.0269 (16)	0.0208 (16)	0.0061 (14)
C18	0.070 (2)	0.075 (2)	0.062 (2)	0.045 (2)	0.0319 (19)	0.0127 (18)
C19	0.069 (2)	0.085 (3)	0.076 (2)	0.045 (2)	0.046 (2)	0.027 (2)
C20	0.057 (2)	0.0581 (19)	0.075 (2)	0.0238 (16)	0.0384 (18)	0.0188 (17)
C21	0.063 (2)	0.0371 (15)	0.0520 (18)	0.0202 (15)	0.0238 (17)	0.0058 (13)
C22	0.0530 (18)	0.0341 (14)	0.0463 (15)	0.0157 (13)	0.0227 (14)	0.0072 (12)
C23	0.053 (2)	0.0530 (18)	0.065 (2)	0.0177 (16)	0.0259 (17)	0.0215 (16)
C24	0.057 (2)	0.071 (2)	0.071 (2)	0.0272 (19)	0.0223 (19)	0.0286 (19)
C25	0.073 (2)	0.0572 (19)	0.0578 (19)	0.0283 (18)	0.0309 (18)	0.0243 (16)
C26	0.059 (2)	0.0432 (16)	0.0530 (17)	0.0185 (14)	0.0315 (16)	0.0100 (13)
C27	0.0513 (18)	0.0420 (15)	0.0513 (17)	0.0184 (14)	0.0232 (15)	0.0091 (13)
C28	0.073 (2)	0.069 (2)	0.083 (3)	0.027 (2)	0.048 (2)	0.031 (2)
O3	0.090 (2)	0.085 (2)	0.167 (3)	0.0562 (18)	0.066 (2)	0.026 (2)
O4	0.0534 (15)	0.0782 (18)	0.093 (2)	0.0271 (14)	0.0242 (14)	0.0129 (15)
C29	0.067 (2)	0.056 (2)	0.105 (3)	0.0312 (19)	0.052 (2)	0.032 (2)
C30	0.062 (2)	0.0472 (17)	0.070 (2)	0.0239 (16)	0.0406 (18)	0.0185 (15)
C31	0.094 (3)	0.054 (2)	0.082 (3)	0.031 (2)	0.057 (2)	0.0113 (18)
C32	0.083 (3)	0.070 (2)	0.059 (2)	0.019 (2)	0.028 (2)	−0.0019 (18)
C33	0.066 (2)	0.072 (2)	0.060 (2)	0.023 (2)	0.0261 (19)	0.0182 (18)
C34	0.059 (2)	0.0522 (17)	0.0575 (18)	0.0235 (16)	0.0329 (17)	0.0241 (15)
C35	0.061 (2)	0.0431 (15)	0.0523 (17)	0.0208 (15)	0.0327 (16)	0.0149 (13)
C36	0.077 (3)	0.076 (2)	0.099 (3)	0.047 (2)	0.049 (2)	0.036 (2)
O5	0.0525 (17)	0.078 (2)	0.153 (3)	0.0089 (16)	0.0260 (19)	0.041 (2)
O6	0.097 (2)	0.139 (3)	0.166 (4)	0.066 (2)	0.096 (3)	0.086 (3)
C37	0.047 (2)	0.091 (3)	0.120 (4)	0.035 (2)	0.035 (3)	0.058 (3)
C38	0.0471 (18)	0.0550 (18)	0.0617 (19)	0.0245 (15)	0.0203 (16)	0.0236 (16)
C39	0.065 (2)	0.061 (2)	0.062 (2)	0.0334 (18)	0.0318 (18)	0.0166 (16)
C40	0.063 (2)	0.0486 (18)	0.073 (2)	0.0169 (17)	0.0267 (19)	0.0072 (17)
C41	0.068 (2)	0.067 (2)	0.084 (3)	0.026 (2)	0.043 (2)	0.033 (2)
C42	0.078 (3)	0.067 (2)	0.062 (2)	0.040 (2)	0.0350 (19)	0.0239 (17)
C43	0.061 (2)	0.0448 (17)	0.0561 (19)	0.0219 (16)	0.0110 (17)	0.0085 (15)
C44	0.143 (5)	0.122 (4)	0.089 (3)	0.075 (4)	0.074 (3)	0.034 (3)
O7	0.205 (4)	0.092 (2)	0.115 (3)	0.074 (3)	0.075 (3)	0.043 (2)
O8	0.168 (3)	0.098 (2)	0.156 (3)	0.074 (2)	0.131 (3)	0.057 (2)

### Geometric parameters (Å, º)

**Table:**

Zn1—N2	2.114 (3)	C22—C27	1.389 (4)
Zn1—O1	2.118 (3)	C23—C24	1.379 (5)
Zn1—N4	2.118 (2)	C23—H23A	0.9300
Zn1—N1	2.129 (3)	C24—C25	1.377 (5)
Zn1—N3	2.138 (3)	C24—H24A	0.9300
Zn1—O2	2.308 (2)	C25—C26	1.377 (5)
Zn1—C21	2.537 (3)	C25—H25A	0.9300
O1—C21	1.267 (4)	C26—C27	1.394 (4)
O2—C21	1.248 (4)	C26—C28	1.504 (5)
N1—C1	1.332 (5)	C27—H27A	0.9300
N1—C5	1.350 (4)	C28—H28A	0.9600
N2—C10	1.335 (5)	C28—H28B	0.9600
N2—C6	1.347 (4)	C28—H28C	0.9600
N3—C11	1.342 (4)	O3—C29	1.207 (4)
N3—C15	1.343 (4)	O4—C29	1.306 (5)
N4—C20	1.339 (4)	O4—H4B	0.8593
N4—C16	1.345 (4)	C29—C30	1.489 (5)
C1—C2	1.368 (6)	C30—C35	1.380 (4)
C1—H1A	0.9300	C30—C31	1.399 (5)
C2—C3	1.367 (7)	C31—C32	1.363 (6)
C2—H2A	0.9300	C31—H31A	0.9300
C3—C4	1.365 (7)	C32—C33	1.360 (6)
C3—H3A	0.9300	C32—H32A	0.9300
C4—C5	1.377 (5)	C33—C34	1.383 (5)
C4—H4A	0.9300	C33—H33A	0.9300
C5—C6	1.479 (5)	C34—C35	1.376 (5)
C6—C7	1.393 (5)	C34—C36	1.496 (5)
C7—C8	1.370 (6)	C35—H35A	0.9300
C7—H7A	0.9300	C36—H36A	0.9600
C8—C9	1.370 (6)	C36—H36B	0.9600
C8—H8A	0.9300	C36—H36C	0.9600
C9—C10	1.377 (5)	O5—C37	1.278 (6)
C9—H9A	0.9300	O6—C37	1.236 (6)
C10—H10A	0.9300	C37—C38	1.505 (5)
C11—C12	1.364 (5)	C38—C39	1.381 (5)
C11—H11A	0.9300	C38—C43	1.382 (5)
C12—C13	1.370 (6)	C39—C40	1.371 (5)
C12—H12A	0.9300	C39—H39A	0.9300
C13—C14	1.378 (5)	C40—C41	1.372 (5)
C13—H13A	0.9300	C40—H40A	0.9300
C14—C15	1.385 (4)	C41—C42	1.373 (5)
C14—H14A	0.9300	C41—H41A	0.9300
C15—C16	1.480 (4)	C42—C43	1.380 (5)
C16—C17	1.385 (4)	C42—C44	1.505 (5)
C17—C18	1.377 (5)	C43—H43A	0.9300
C17—H17A	0.9300	C44—H44A	0.9600
C18—C19	1.363 (5)	C44—H44B	0.9600
C18—H18A	0.9300	C44—H44C	0.9600
C19—C20	1.369 (5)	O7—H7B	0.8596
C19—H19A	0.9300	O7—H7C	0.8764
C20—H20A	0.9300	O8—H8B	0.8787
C21—C22	1.499 (4)	O8—H8C	0.8502
C22—C23	1.374 (5)		
			
N2—Zn1—O1	147.48 (10)	C20—C19—H19A	120.5
N2—Zn1—N4	99.00 (11)	N4—C20—C19	122.5 (3)
O1—Zn1—N4	95.44 (10)	N4—C20—H20A	118.7
N2—Zn1—N1	77.29 (11)	C19—C20—H20A	118.7
O1—Zn1—N1	91.68 (10)	O2—C21—O1	121.5 (3)
N4—Zn1—N1	171.37 (10)	O2—C21—C22	119.7 (3)
N2—Zn1—N3	108.16 (11)	O1—C21—C22	118.8 (3)
O1—Zn1—N3	103.36 (10)	O2—C21—Zn1	65.07 (18)
N4—Zn1—N3	77.35 (10)	O1—C21—Zn1	56.42 (17)
N1—Zn1—N3	96.28 (11)	C22—C21—Zn1	175.2 (3)
N2—Zn1—O2	91.52 (10)	C23—C22—C27	119.3 (3)
O1—Zn1—O2	59.28 (9)	C23—C22—C21	120.3 (3)
N4—Zn1—O2	90.36 (9)	C27—C22—C21	120.4 (3)
N1—Zn1—O2	97.48 (10)	C22—C23—C24	120.3 (3)
N3—Zn1—O2	158.07 (9)	C22—C23—H23A	119.8
N2—Zn1—C21	119.80 (11)	C24—C23—H23A	119.8
O1—Zn1—C21	29.91 (10)	C25—C24—C23	119.6 (3)
N4—Zn1—C21	93.20 (10)	C25—C24—H24A	120.2
N1—Zn1—C21	95.40 (10)	C23—C24—H24A	120.2
N3—Zn1—C21	132.02 (11)	C26—C25—C24	121.8 (3)
O2—Zn1—C21	29.38 (9)	C26—C25—H25A	119.1
C21—O1—Zn1	93.7 (2)	C24—C25—H25A	119.1
C21—O2—Zn1	85.5 (2)	C25—C26—C27	117.7 (3)
C1—N1—C5	118.8 (3)	C25—C26—C28	121.2 (3)
C1—N1—Zn1	125.4 (3)	C27—C26—C28	121.1 (3)
C5—N1—Zn1	115.8 (2)	C22—C27—C26	121.2 (3)
C10—N2—C6	118.9 (3)	C22—C27—H27A	119.4
C10—N2—Zn1	125.7 (2)	C26—C27—H27A	119.4
C6—N2—Zn1	115.4 (2)	C26—C28—H28A	109.5
C11—N3—C15	118.3 (3)	C26—C28—H28B	109.5
C11—N3—Zn1	126.8 (2)	H28A—C28—H28B	109.5
C15—N3—Zn1	114.8 (2)	C26—C28—H28C	109.5
C20—N4—C16	118.7 (3)	H28A—C28—H28C	109.5
C20—N4—Zn1	125.3 (2)	H28B—C28—H28C	109.5
C16—N4—Zn1	115.5 (2)	C29—O4—H4B	113.6
N1—C1—C2	122.9 (5)	O3—C29—O4	123.7 (4)
N1—C1—H1A	118.6	O3—C29—C30	122.9 (4)
C2—C1—H1A	118.6	O4—C29—C30	113.3 (3)
C3—C2—C1	118.3 (5)	C35—C30—C31	118.3 (3)
C3—C2—H2A	120.8	C35—C30—C29	121.4 (3)
C1—C2—H2A	120.8	C31—C30—C29	120.4 (3)
C4—C3—C2	119.7 (4)	C32—C31—C30	119.5 (3)
C4—C3—H3A	120.2	C32—C31—H31A	120.2
C2—C3—H3A	120.2	C30—C31—H31A	120.2
C3—C4—C5	119.8 (4)	C33—C32—C31	121.1 (3)
C3—C4—H4A	120.1	C33—C32—H32A	119.5
C5—C4—H4A	120.1	C31—C32—H32A	119.5
N1—C5—C4	120.6 (4)	C32—C33—C34	121.1 (4)
N1—C5—C6	114.8 (3)	C32—C33—H33A	119.4
C4—C5—C6	124.7 (4)	C34—C33—H33A	119.4
N2—C6—C7	120.8 (4)	C35—C34—C33	117.6 (3)
N2—C6—C5	116.7 (3)	C35—C34—C36	121.1 (3)
C7—C6—C5	122.6 (3)	C33—C34—C36	121.2 (3)
C8—C7—C6	119.1 (4)	C34—C35—C30	122.3 (3)
C8—C7—H7A	120.5	C34—C35—H35A	118.8
C6—C7—H7A	120.5	C30—C35—H35A	118.8
C9—C8—C7	120.3 (4)	C34—C36—H36A	109.5
C9—C8—H8A	119.9	C34—C36—H36B	109.5
C7—C8—H8A	119.9	H36A—C36—H36B	109.5
C8—C9—C10	117.9 (4)	C34—C36—H36C	109.5
C8—C9—H9A	121.1	H36A—C36—H36C	109.5
C10—C9—H9A	121.1	H36B—C36—H36C	109.5
N2—C10—C9	123.1 (4)	O6—C37—O5	125.6 (4)
N2—C10—H10A	118.4	O6—C37—C38	118.8 (4)
C9—C10—H10A	118.4	O5—C37—C38	115.6 (5)
N3—C11—C12	123.3 (3)	C39—C38—C43	118.8 (3)
N3—C11—H11A	118.4	C39—C38—C37	121.4 (4)
C12—C11—H11A	118.4	C43—C38—C37	119.8 (4)
C11—C12—C13	118.3 (4)	C40—C39—C38	119.6 (3)
C11—C12—H12A	120.8	C40—C39—H39A	120.2
C13—C12—H12A	120.8	C38—C39—H39A	120.2
C12—C13—C14	119.8 (4)	C39—C40—C41	120.3 (3)
C12—C13—H13A	120.1	C39—C40—H40A	119.9
C14—C13—H13A	120.1	C41—C40—H40A	119.9
C13—C14—C15	118.8 (3)	C40—C41—C42	121.8 (4)
C13—C14—H14A	120.6	C40—C41—H41A	119.1
C15—C14—H14A	120.6	C42—C41—H41A	119.1
N3—C15—C14	121.4 (3)	C41—C42—C43	117.0 (3)
N3—C15—C16	116.2 (3)	C41—C42—C44	122.0 (4)
C14—C15—C16	122.4 (3)	C43—C42—C44	121.0 (4)
N4—C16—C17	121.2 (3)	C42—C43—C38	122.4 (3)
N4—C16—C15	115.8 (2)	C42—C43—H43A	118.8
C17—C16—C15	123.0 (3)	C38—C43—H43A	118.8
C18—C17—C16	119.0 (3)	C42—C44—H44A	109.5
C18—C17—H17A	120.5	C42—C44—H44B	109.5
C16—C17—H17A	120.5	H44A—C44—H44B	109.5
C19—C18—C17	119.5 (3)	C42—C44—H44C	109.5
C19—C18—H18A	120.3	H44A—C44—H44C	109.5
C17—C18—H18A	120.3	H44B—C44—H44C	109.5
C18—C19—C20	119.0 (3)	H7B—O7—H7C	109.1
C18—C19—H19A	120.5	H8B—O8—H8C	103.7
			
N2—Zn1—N1—C1	−179.7 (3)	C11—N3—C15—C14	−1.4 (5)
O1—Zn1—N1—C1	31.2 (3)	Zn1—N3—C15—C14	177.7 (3)
N3—Zn1—N1—C1	−72.5 (3)	C11—N3—C15—C16	178.4 (3)
O2—Zn1—N1—C1	90.4 (3)	Zn1—N3—C15—C16	−2.4 (3)
N2—Zn1—N1—C5	1.4 (2)	C13—C14—C15—N3	0.8 (5)
O1—Zn1—N1—C5	−147.7 (2)	C13—C14—C15—C16	−179.1 (3)
N3—Zn1—N1—C5	108.7 (2)	C20—N4—C16—C17	−1.3 (5)
O2—Zn1—N1—C5	−88.4 (2)	Zn1—N4—C16—C17	171.1 (2)
N4—Zn1—N2—C10	9.3 (3)	C20—N4—C16—C15	−179.4 (3)
O1—Zn1—N2—C10	−105.9 (3)	Zn1—N4—C16—C15	−7.0 (3)
N1—Zn1—N2—C10	−178.6 (3)	N3—C15—C16—N4	6.3 (4)
N3—Zn1—N2—C10	88.9 (3)	C14—C15—C16—N4	−173.8 (3)
O2—Zn1—N2—C10	−81.3 (3)	N3—C15—C16—C17	−171.8 (3)
N4—Zn1—N2—C6	−173.7 (2)	C14—C15—C16—C17	8.1 (5)
O1—Zn1—N2—C6	71.0 (3)	N4—C16—C17—C18	0.9 (5)
N1—Zn1—N2—C6	−1.7 (2)	C15—C16—C17—C18	178.9 (3)
N3—Zn1—N2—C6	−94.2 (2)	C16—C17—C18—C19	−0.1 (5)
O2—Zn1—N2—C6	95.7 (2)	C17—C18—C19—C20	−0.2 (6)
N2—Zn1—N3—C11	82.7 (3)	C16—N4—C20—C19	1.0 (5)
N4—Zn1—N3—C11	178.1 (3)	Zn1—N4—C20—C19	−170.6 (3)
O1—Zn1—N3—C11	−89.2 (3)	C18—C19—C20—N4	−0.3 (6)
N1—Zn1—N3—C11	4.0 (3)	Zn1—O2—C21—O1	0.4 (3)
O2—Zn1—N3—C11	−124.5 (3)	Zn1—O2—C21—C22	−179.8 (2)
N2—Zn1—N3—C15	−96.4 (2)	Zn1—O1—C21—O2	−0.4 (3)
N4—Zn1—N3—C15	−0.9 (2)	Zn1—O1—C21—C22	179.8 (2)
O1—Zn1—N3—C15	91.8 (2)	O2—C21—C22—C23	−170.3 (3)
N1—Zn1—N3—C15	−175.0 (2)	O1—C21—C22—C23	9.6 (4)
O2—Zn1—N3—C15	56.4 (4)	O2—C21—C22—C27	10.0 (4)
N2—Zn1—N4—C20	−77.0 (3)	O1—C21—C22—C27	−170.2 (3)
O1—Zn1—N4—C20	73.7 (3)	C27—C22—C23—C24	−1.1 (5)
N3—Zn1—N4—C20	176.2 (3)	C21—C22—C23—C24	179.1 (3)
O2—Zn1—N4—C20	14.6 (3)	C22—C23—C24—C25	1.0 (5)
N2—Zn1—N4—C16	111.1 (2)	C23—C24—C25—C26	0.1 (6)
O1—Zn1—N4—C16	−98.1 (2)	C24—C25—C26—C27	−1.0 (5)
N3—Zn1—N4—C16	4.4 (2)	C24—C25—C26—C28	179.8 (3)
O2—Zn1—N4—C16	−157.3 (2)	C23—C22—C27—C26	0.2 (4)
C5—N1—C1—C2	0.0 (6)	C21—C22—C27—C26	180.0 (3)
Zn1—N1—C1—C2	−178.9 (3)	C25—C26—C27—C22	0.8 (4)
N1—C1—C2—C3	0.7 (7)	C28—C26—C27—C22	−179.9 (3)
C1—C2—C3—C4	0.1 (7)	O3—C29—C30—C35	177.8 (4)
C2—C3—C4—C5	−1.4 (7)	O4—C29—C30—C35	−2.7 (5)
C1—N1—C5—C4	−1.3 (5)	O3—C29—C30—C31	−3.8 (6)
Zn1—N1—C5—C4	177.6 (2)	O4—C29—C30—C31	175.7 (3)
C1—N1—C5—C6	−179.9 (3)	C35—C30—C31—C32	1.8 (5)
Zn1—N1—C5—C6	−1.0 (3)	C29—C30—C31—C32	−176.6 (4)
C3—C4—C5—N1	2.1 (5)	C30—C31—C32—C33	−0.9 (6)
C3—C4—C5—C6	−179.5 (4)	C31—C32—C33—C34	0.2 (6)
C10—N2—C6—C7	−0.6 (5)	C32—C33—C34—C35	−0.4 (5)
Zn1—N2—C6—C7	−177.8 (2)	C32—C33—C34—C36	179.9 (4)
C10—N2—C6—C5	178.8 (3)	C33—C34—C35—C30	1.4 (5)
Zn1—N2—C6—C5	1.7 (3)	C36—C34—C35—C30	−178.9 (3)
N1—C5—C6—N2	−0.5 (4)	C31—C30—C35—C34	−2.1 (5)
C4—C5—C6—N2	−179.0 (3)	C29—C30—C35—C34	176.3 (3)
N1—C5—C6—C7	179.0 (3)	O6—C37—C38—C39	7.0 (6)
C4—C5—C6—C7	0.5 (5)	O5—C37—C38—C39	−173.4 (3)
N2—C6—C7—C8	−0.3 (5)	O6—C37—C38—C43	−172.4 (4)
C5—C6—C7—C8	−179.8 (3)	O5—C37—C38—C43	7.2 (5)
C6—C7—C8—C9	0.6 (6)	C43—C38—C39—C40	0.8 (5)
C7—C8—C9—C10	0.1 (6)	C37—C38—C39—C40	−178.7 (3)
C6—N2—C10—C9	1.4 (5)	C38—C39—C40—C41	0.9 (6)
Zn1—N2—C10—C9	178.2 (3)	C39—C40—C41—C42	−1.1 (6)
C8—C9—C10—N2	−1.1 (6)	C40—C41—C42—C43	−0.3 (6)
C15—N3—C11—C12	0.7 (5)	C40—C41—C42—C44	−180.0 (4)
Zn1—N3—C11—C12	−178.3 (3)	C41—C42—C43—C38	1.9 (5)
N3—C11—C12—C13	0.6 (6)	C44—C42—C43—C38	−178.3 (4)
C11—C12—C13—C14	−1.3 (7)	C39—C38—C43—C42	−2.2 (5)
C12—C13—C14—C15	0.6 (6)	C37—C38—C43—C42	177.2 (3)

### Hydrogen-bond geometry (Å, º)

**Table:**

*D*—H···*A*	*D*—H	H···*A*	*D*···*A*	*D*—H···*A*
O4—H4*B*···O5^i^	0.86	1.63	2.492 (5)	175
O7—H7*B*···O5^ii^	0.86	2.45	3.028 (6)	125
O7—H7*C*···O8	0.88	2.14	2.938 (6)	151
O8—H8*B*···O6	0.88	2.10	2.973 (5)	178
O8—H8*C*···O6^ii^	0.85	2.05	2.871 (6)	163
C7—H7*A*···O2^iii^	0.93	2.45	3.234 (5)	142
C17—H17*A*···O1^iv^	0.93	2.44	3.297 (5)	152
C18—H18*A*···O8	0.93	2.47	3.280 (7)	146

Symmetry codes: (i) *x*−1, *y*, *z*−1; (ii) −*x*+2, −*y*+1, −*z*+1; (iii) −*x*+2, −*y*+2, −*z*+2; (iv) −*x*+1, −*y*+1, −*z*+1.
